# Promoting Pre-service Teacher Students’ Learning Engagement: Design-Based Research in a Flipped Classroom

**DOI:** 10.3389/fpsyg.2022.810275

**Published:** 2022-02-21

**Authors:** Jianjun Gu, Lin Tang, Xiaohong Liu, Jinlei Xu

**Affiliations:** School of Education Science, Nanjing Normal University, Nanjing, China

**Keywords:** learning engagement, flipped classroom, design-based research, pre-service teacher student, learning effectiveness

## Abstract

Students’ learning engagement is recognized as one of the main components of effective instruction and a necessary prerequisite for learning, but students’ learning engagement in flipped classroom poses some pedagogical challenges. This study aimed to promote students’ learning engagement via the flipped classroom approach. Design-based research (DBR) was adopted in this study to conduct an experiment involving three iterations in a Modern Educational Technology (MET) course in a Chinese university. The participants included 36 third-year pre-service teacher undergraduates. Classroom observations and a learning engagement questionnaire were used to measure the effectiveness of the flipped instruction in terms of students’ learning engagement. Data analysis applied descriptive statistics, ANOVA, and paired samples *t* tests. The results showed that after three rounds of iterative experiments, students’ learning engagement (behavioral, cognitive, and emotional) significantly improved. Several principles are provided as guidelines for instructors to implement flipped classroom approach to promote students’ learning engagement.

## Introduction

Flipped classroom, as a hybrid teaching method, has gained wide popularity in higher education around the world, with the aim of improving student achievement by promoting their learning engagement and improving their learning experience (Bossaer et al., 2016; Chiang, 2017; Day, 2018). Students’ learning engagement is recognized as one of the main components of effective instruction (Barkley, 2010), and is a necessary prerequisite for learning (Fredricks et al., 2004; Guo et al., 2014). However, although much evidence supports that flipped classrooms play a positive role in improving students’ academic achievement (e.g., Lo et al., 2017; Alten et al., 2019), challenges of promoting students’ learning engagement in this instruction approach are still to be completely solved. For example, some studies have found that students’ academic achievement improved in flipped classrooms, but some key indicators of students’ learning engagement have not been improved (Strayer, 2012), such as interest and satisfaction (Fredricks et al., 2004), which even gradually declined in the process (Sickle, 2016; Alten et al., 2019). However, previous studies have focused on the impact of flipped classrooms and traditional classrooms on students’ learning engagement (e.g., Baepler et al., 2014; Elmaadaway, 2018; Subramaniam and Muniandy, 2019), rather than investigating the performance of students’ learning engagement in flipped classrooms to promote their continuous engagement in study activities. Only a few studies have discussed improving students’ learning engagement in flipped classrooms (Yoon et al., 2018; Lo and Hew, 2021). Therefore, there is still a lack of clear “how to” lists related to designing effective flipped classrooms (Wang, 2017). Teachers need useful instructional strategies for guiding the design and implementation of flipped classrooms for promoting students’ learning engagement, such as effective pre-class learning guidance (Stoltzfus, 2016) and active classroom communication (Graziano, 2017). Teachers do not have sufficient effective strategies to implement flipped classrooms, which may reduce the advantages of flipped classrooms (Karabulut-Ilgu et al., 2018; Lo et al., 2018). Providing more useful teaching strategies for teachers requires understanding how to design effective flipped instruction to promote students’ learning engagement, and how to support students’ learning engagement. In addition, it is worth noticing that some relevant studies only focused on one or two dimensions of learning engagement when discussing how to improve students’ learning engagement in flipped classrooms, such as students’ cognitive engagement (Huang et al., 2019; Lo and Hew, 2020; Wu et al., 2020), and behavioral engagement (Hodgson et al., 2017; Wang, 2017; Lai, 2021), but they have seldom carried out comprehensive investigations of students’ learning engagement (Lo and Hew, 2021). Therefore, this study aimed to comprehensively explore students’ learning engagement in a flipped classroom to make up for the shortcomings of the above research.

In addition, previous studies have investigated the impact of flipped classrooms on students’ cognitive performance, such as satisfaction, motivation, and self-efficacy (Abeysekera and Dawson, 2015; Yilmaz, 2017; Chou, 2018; Lag and Sæle, 2019; Murillo-Zamorano et al., 2019; Zheng et al., 2020; Debba and Yldz, 2021). However, there were usually fewer studies on courses aimed at training students’ skills and strategies (Elmaadaway, 2018; Xu et al., 2019; Lin et al., 2021). Moreover, flipped classrooms applied in technical courses face some challenges (Davies et al., 2013; Lin, 2021). For example, Al-Samarraie et al. (2020) indicated that there is a lack of immediate feedback and time for practical practice when conducting the flipped classroom approach in the software technology course. Therefore, this study aims to explore how to promote students’ learning engagement in a technological course with the flipped classroom approach. A Modern Educational Technology (MET) course was chosen as the case study in the current study. MET is a required course for pre-service teacher students in China, and aims to train them to master auxiliary knowledge and skills in future teaching and to improve their skills of digital teaching. Existing studies have shown that the flipped classroom approach can improve the overall academic achievement of students in this course (Liu, 2017; Zhao et al., 2021b). However, the investigation of students’ learning engagement in the MET course is still lacking. This research further explored the performance of students’ learning engagement in the MET course to fill this gap in the literature.

## Literature Review

### Flipped Classrooms

Flipped classrooms are considered as a student-centered teaching method that advocates students’ learning engagement and active learning (Steen-Utheim and Foldnes, 2018). In the flipped classroom, the content that needs to be taught in the traditional teaching mode is transferred to outside the classroom (Zhang, 2018; Wang, 2019). Students should complete the learning of the course content outside of class, and then conduct collaborative learning and reflection with group members in class to further integrate and build their knowledge systems (Kim et al., 2014; Wang, 2019). The flipped classroom approach emphasizes the idea of problem solving in the classroom, which is associated with many instructional approaches, such as active learning (Silberman, 1996), collaborative learning (Dillenbourg, 1999), and problem-based learning (Hmelo-Silver et al., 2007). These are just some of the many instantiations of constructivism, a popular learning theory that emphasizes learners constructing their own understanding and perceptions of things by experiencing and reflecting on those experiences (Piaget, 1950; Fosnot, 2005). Consistent with the constructivist learning theory, in flipped classrooms learners are regarded as knowledge constructors and active agents in the learning activity (King, 1993). There is no fixed mode for flipped classrooms, but the core elements include advanced content, educators’ awareness of students’ understanding, and higher-order learning during class time (O’Flaherty and Phillips, 2015), while the core idea is that teachers’ instruction is combined with constructivist learning theory, and students’ learning extends beyond the classroom where they can learn at their own pace and receive personalized instruction (Davies et al., 2013). The flipped classroom approach requires students to study the learning content before class independently, and students can receive more personalized guidance from teachers in class (Xu and Feng, 2017). The theory of individualized instruction refers to the teaching strategies adopted in the learning process to meet the needs of individual learners, and to ensure that they receive appropriate support or feedback (Ferster, 1968). Therefore, constructivist learning and personalized teaching theories provide theoretical support for flipped classrooms.

The flipped classroom approach has been adopted for a growing number of higher education subjects such as psychology (Roehling et al., 2017), mathematics (Lo, 2017), medicine (Gilboy et al., 2015), and biochemistry (Ojennus, 2016). Many studies have reported that the flipped classroom approach can promote students’ learning engagement (e.g., Baepler et al., 2014; Kong, 2014; Roach, 2014; Saulnier, 2015). The findings above show that students can achieve better learning results in flipped classrooms than in traditional classrooms.

However, flipped classrooms are not always effective. Some research reports have claimed that flipped classrooms did not significantly improve students’ learning effects compared with traditional classrooms (Sun et al., 2017; Tse et al., 2019). For example, it has been reported in pharmaceutical education (Gillette et al., 2018), engineering education (Cheng et al., 2019), and a technology literacy course (Sommer and Ritzhaupt, 2018). The flipped classroom model has some potential challenges, which may have a negative impact on students’ learning engagement. For example, students need to watch instructional videos and complete practice tests before lecture-based classes in flipped classrooms. The preparation work may lead to a greater workload for students (Huang et al., 2020), and even affect students’ learning engagement (Fredricks et al., 2004). Specifically, improper selection and organization of pre-class learning materials, even late delivery of pre-class materials to students, may hinder students’ active performance (Shahnama et al., 2021). Moreover, pre-class quizzes can also cause anxiety and stress for some students (Tune et al., 2013; Alavi et al., 2016), and some students even argue that quizzes were not helpful for improving their learning performance (Broman and Johnels, 2019). As often reported in the literature, students usually fail to complete their autonomous learning tasks before class (Chuang et al., 2016; Lo et al., 2017), which significantly influences their active learning engagement in the face-to-face class learning activities (Mason et al., 2013; Kim et al., 2014). Some researchers have pointed out that it is difficult for students to fully engage in group cooperation in flipped learning (Halili et al., 2014; Kim et al., 2014). The challenges of group cooperation include responsibilities and effective discussions (Popov et al., 2012; Graziano, 2017). There are also some students who cannot adapt to the new method and feel anxious or resistant (Chen et al., 2014; Porcaro et al., 2016). On the other hand, it can be difficult for teachers to effectively combine the face-to-face part with the extracurricular part, resulting in a lack of continuity in teaching (Lee, 2017). If there is no close connection between the face-to-face and the extracurricular parts in flipped classrooms, it may distract students from engaging in the learning activities (Elen and Clarebout, 2001; Buerck et al., 2003). Teachers may also be challenged by engaging in more face-to-face communication with students (Rodriguez, 2016). Moreover, because of the large number of students, it may be difficult for teachers to give personalized guidance to all students in class (Clark et al., 2016). At the same time, teachers often fail to give immediate feedback when students ask questions during online lectures (Cha and Kim, 2016; Clark et al., 2016). Therefore, these studies have shown that there are still challenges in designing and implementing an effective flipped classroom, but previous studies on learning engagement mainly focused on comparing flipped classrooms with traditional classrooms (e.g., Baepler et al., 2014; Elmaadaway, 2018; Subramaniam and Muniandy, 2019). Further research is thus needed to determine which effective design elements in flipped classrooms enable students to perform better in terms of their learning engagement.

### Learning Engagement

Students’ learning engagement is an important aspect of teaching activities, and is considered to be the basic structure of providing quality education, and a factor associated with academic success (Fisher et al., 2018). Learning engagement is widely defined as students’ positive performance in three aspects: cognition, behavior, and emotion (Fredricks et al., 2004). Meyer and Turner (2002) pointed out that behavioral engagement is represented by persistence and endeavor in learning activities. Cognitive engagement is the psychological effort that students put into their learning, involving self-regulation and metacognitive behaviors (Fredricks et al., 2004; Chiu, 2021). Emotional engagement involves the learner’s general emotional responses to learning, such as interest, enjoyment, satisfaction, frustration, and social interaction (Fredricks et al., 2004). Based on these dimensions, many related studies have been carried out in flipped classrooms.

Since behavioral engagement is easier to observe, many scholars have studied behavioral engagement in flipped classrooms (e.g., Hodgson et al., 2017; Wang, 2017; Huang et al., 2019; Lai, 2021). In recent studies, there were also researchers who divided behavioral engagement in flipped classrooms into in-class and extracurricular learning activities (Wang, 2019; Ranellucci et al., 2021). However, in extracurricular activities, one of the major challenges of flipped classrooms is that students are not adequately prepared for pre-class learning activities (Akçayır and Akçayır, 2018) which are the key to the success of flipped classrooms, facilitating students’ face-to-face learning engagement (Rahman et al., 2015; Yilmaz and Baydas, 2017). Therefore, attention must be paid to the pre-class preparation activities in flipped classrooms, which will directly affect the learning engagement in class. What’s more, existing studies have shown that gamification can enhance students’ cognitive engagement in flipped classrooms (Huang et al., 2019; Lo and Hew, 2020). Emotional engagement is related to cognition and behavior. Increasing evidence has found that students who lack emotional engagement in learning will gradually decrease their cognitive and behavioral engagement (Archambault et al., 2009; Hirschfield and Gasper, 2011). Therefore, the research on improving students’ emotional engagement has become increasingly important. Some researchers have proved that game-based learning tasks can effectively improve students’ emotional engagement (Ninaus et al., 2019; Zainuddin et al., 2020). Teachers’ emotional support is also an important way to promote students’ emotional engagement (Rimm-Kaufman et al., 2014). These studies provide theoretical and practical supports for this study to promote students’ learning engagement in a flipped classroom.

However, studies on different dimensions of students’ learning engagement in flipped classrooms are still insufficient, and only a few have considered the three dimensions of learning engagement, namely cognition, behavior, and emotion (Lo and Hew, 2021). On the one hand, some studies regarded learning engagement as a whole, failed to provide a clear definition of learning engagement, and lacked in-depth exploration of different dimensions of learning engagement (Muir and Geiger, 2016; Muir, 2017). On the other hand, some studies only focused on promoting one or two dimensions of learning engagement in flipped classrooms (e.g., Wang, 2017; Huang et al., 2019; Lo and Hew, 2020; Lai, 2021). This study focused on the three dimensions to further explore effective flipped instruction that promotes students’ learning engagement.

In addition, it was found through a literature review that most studies used self-reported methods to measure students’ learning engagement in flipped classrooms (e.g., Gilboy et al., 2015; Henrie et al., 2015; Elmaadaway, 2018; Lundin et al., 2018; Bond, 2020). This may raise questions about the validity of using self-reporting on its own (Hodgson et al., 2017). Although some studies combined interviews or observations for qualitative assessment (e.g., Jo et al., 2018; Cevikbas and Kaiser, 2021), the disadvantage is that it is not clear how to systematically measure or define students’ learning engagement, which makes it difficult to assess. In addition, Henrie et al. (2015) reported that the existing quantitative observations measure the level of students’ learning engagement focused on a variety of frequency indicators, such as students’ clickstreams (Seo et al., 2021). The weakness of these studies is that they may not adequately measure cognitive and emotional engagement. Therefore, self-reported and non-self-reported data collection methods were used to measure students’ learning engagement in this study, which may help draw accurate conclusions about students’ learning engagement. Importantly, this study chose a quantitative classroom observation tool that differed from previous ones as it provides a clear definition of the three dimensions of students’ learning engagement in observation. This study attempted to expand the impact of quantitative observation on students’ learning engagement.

### Design-Based Research

Design-based research (DBR), otherwise referred to as educational design research, is defined as “a systematic but flexible methodology designed to improve educational practices based on collaboration between researchers and practitioners in real-world settings and to lead to context-sensitive design principles and theories through iterative analysis, design, development, and implementation” (Wang and Hannafin, 2005). Reeves (2006) believed that design-based research should follow four phases: (1) analyze practical problems, (2) develop solutions based on existing knowledge, (3) evaluate solutions in practice, and (4) reflect on the resulting design principles. The advantage of DBR is that it narrows the gap between theory and practice in research through continuous iterative cycles. DBR can be purposefully explored in a variety of learning environments (Sandoval, 2013), with some research extending to flipped classrooms (Hung, 2017; Meyer et al., 2018). Due to the iterative advantages of DBR, some studies used DBR to modify and improve the design of flipped classrooms (Chiang and Chen, 2017; Hung, 2017). For example, one study proposed a teaching strategy framework for promoting active and reflective learning in flipped classrooms, which has been continuously improved through DBR (Fauzi and Hussain, 2016). Recently, some studies have verified their propositions and rationales in real flipped classrooms based on the DBR method (Froehlich, 2018; Atkinson et al., 2020; Schallert et al., 2021; Zhao et al., 2021a). Therefore, design-based research has the potential to be further implemented in flipped classrooms. However, only a few studies have used the DBR approach to analyze students’ learning engagement in flipped classrooms (Lo and Hew, 2021).

This study aimed to not only understand what is happening in flipped classrooms, but also to understand how learning engagement can be promoted in the application of the flipped classroom approach, especially when students are faced with real learning tasks. The DBR approach provides proof of not only what works, but also of how and why something works (Bakker and Eerde, 2015). Therefore, DBR was used in this study as a methodological basis to design effective flipped instruction to promote pre-service teacher students’ learning engagement. In addition, the flipped instruction was continuously perfected in real practice to improve the effectiveness of this instruction.

### Research Questions

This study aimed to promote teacher students’ learning engagement in a flipped classroom. The DBR approach was used to design and refine flipped instruction that supports students’ learning engagement. The following two questions provided direction for this study:

1. Did students’ learning engagement (behavioral, cognitive, and emotional) improve as a result of the revisions of the three rounds of flipped instruction?
2. What comprises effective flipped instruction in the MET course?

## Methods

### Participants

There were 36 participants, an intact class of third-year pre-service teacher students, receiving the flipped classroom instruction. They were all about 20 years old. They enrolled in the MET course at a university in China during the spring of 2021 to prepare for their future teaching careers. The whole process was instructed by the same teacher. Before the experiment, participants were told that they were taking part in an educational study, and their learning process would be videotaped. The information they provided was anonymous. This study received the consent of all participants.

### Measurement

Two data collection tools were used to assess students’ learning engagement in response to the above research questions, including classroom observation and the learning engagement questionnaire. The method of process assessments and summative assessments was adopted to measure the pre-service teacher students’ learning engagement. Each iteration of the experiment evaluated students’ learning engagement through classroom observation. Pre- and post-intervention survey questionnaires were given to students before and after the experiment to measure their learning engagement.

**FIGURE 1:**
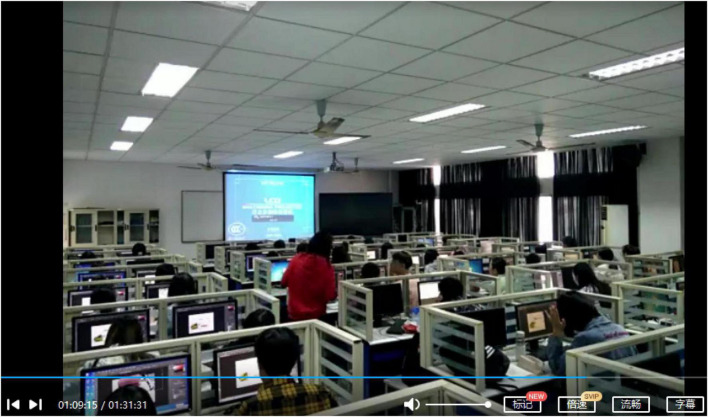
Video recording of classroom teaching.

The quantitative classroom observation framework adopted in this study was developed by Alicea et al. (2016), and was divided into three major domains: behavioral engagement, cognitive engagement, and emotional engagement. These areas are measured by 13 specific dimensions. These items were evaluated on a continuum (on a 5-point scale ranging from 1 = *low engagement* to 5 = *high engagement*) by watching recorded videos (see Figure 1). Two experienced experts and a university professor examined the effectiveness of the classroom observation framework, and provided feedback. Based on the experts’ advice and combined with the characteristics of the participants in this study, two items were deleted and the observation framework was modified to 11 items (see Table 1). The raters in this study consisted of two researchers and a teacher in the field of educational technology. To ensure the interrater reliability, all raters who participated in data collection were trained to strictly follow scoring guidelines for each fragment observed. In the 90-min lesson, according to the observation framework, three raters scored at the 10th minute, 30th minute, 50th minute, 70th minute, and 90th minute. Therefore, in this study, a total score was calculated for each item in a lesson based on the average score of the five observation segments in the 90-min lesson; for instance, (attentiveness 1 + attentiveness 2 + …+ attentiveness 5)/5 = total score of “attentiveness” in a lesson. Finally, a composite score of each domain was established based on the average score of the domain in all observation segments. Interrater reliability was more than 80% according to a reliability test (La Paro et al., 2004). In addition, Cronbach’s alpha (Pallant, 2007) estimation of the three-factor structural scale of the classroom observation framework showed good consistency for the domains “behavioral engagement” (α = 0.77), “cognitive engagement” (α = 0.86), and “emotional engagement” (α = 0.86), showing that the classroom observation framework had good reliability. Table 1 provides an overview of the domains, dimensions, and standards descriptions.

**TABLE 1:** Description of the classroom observation framework.

Domain	Dimension	Description
Behavioral engagement	Attentiveness	Most of the students exhibit attentive body language
	Rule compliance	Most students follow the instructor’s prompts
	Engaging	The instructor shows enthusiasm and interest when engaging with students
	Learning organization	The materials and discussion are related to clear learning objectives
	Classroom management	There are no disruptions in the classroom
Emotional engagement	Comfort	Interactions in the class are relaxed, empathetic, and warm
	Validation	Class members praise and support each other’s efforts appropriately
	Fairness/Inclusion	The instructor encourages participation of multiple diverse participants, and the classroom has a “democratic” atmosphere
Cognitive engagement	Curiosity	The students perform activities that generate how and why questions which are linked to critical thinking
	Content level	Content falls within the zone of proximal development
	Student balance of involvement	Participation in the cognitive tasks (discussion, group work) is evenly distributed among students in the class

The learning engagement questionnaire used in this study was developed by Elmaadaway (2018). Participants completed this structured questionnaire before and after the entire course. The questionnaire was divided into three parts with 25 questions, involving behavioral engagement (10 items), cognitive engagement (7 items), and emotional engagement (8 items). These items were evaluated on a 5-point Likert scale (5 = *strongly agree*, 4 = *agree*, 3 = *neutral*, 2 = *disagree*, 1 = *strongly disagree*). To accommodate the participants who are non-native English speakers in this study, the questionnaire was also translated and reviewed by two experts in the field of higher education. Then, to ensure the accuracy and clarity of the questionnaire in Chinese, three college students were invited to answer each item in the questionnaire one by one and to give detailed feedback. The Cronbach’s alpha values of the three dimensions were 0.80 (behavioral engagement), 0.82 (cognitive engagement), and 0.86 (emotional engagement), indicating their reliability.

### Selection of Instructional Content

Modern Educational Technology is a common course in many teacher education universities in China, and is aimed at cultivating and improving the technical literacy of pre-service teachers and students, that is, the ability to understand and apply technical tools in teaching (Sommer and Ritzhaupt, 2018). The MET course focuses on procedural knowledge, and mainly helps students to master some operational skills, such as mastery of Adobe Photoshop, Adobe Premiere, Camtasia studios, and so on, which are the basic skills future educators should master. Four topics of the Adobe Premiere (PR) module were selected for the experiments, namely video clip (topic 1), video transition effect (topic 2), video effects application (topic 3), and subtitle adding and multi-track editing (topic 4). This module includes an introduction to the basic concepts of Adobe Premiere, with a focus on practical exercises. The teacher used a number of techniques for instructional demonstrations in the classroom, with the goal of having students learn to generate and edit specific videos.

### Design of the Three-Round Flipped Instruction to Promote Students’ Learning Engagement

According to the design-based research approach, four interlinked phases were adopted to design flipped instruction promoting students’ learning engagement: (1) define and design flipped instruction, (2) implement and verify flipped instruction, (3) analyze and evaluate flipped instruction, and (4) improve and optimize flipped instruction. The entire DBR process is iteratively nested, rather than being in a linear sequence. Three iterations were implemented, and the flipped instruction was constantly improved.

**FIGURE 2:**
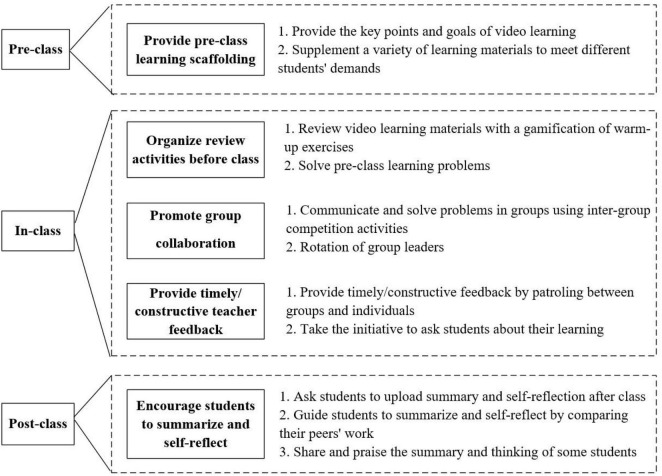
Version 3 of the flipped instruction to promote students’ learning engagement.

The constructivist learning theory (Fosnot, 2005) was used to help guide the development of this preliminary framework. Also, the individualized development of students should be taken into account and reflected on when deciding to try a new instructional design. Thus, individualized instruction theory (Ferster, 1968) was applicable. The instructional design was first tested in class in a prototype form and then refined over three iterations, resulting in some flipped instruction that promoted students’ learning engagement. In the first round, five design principles and version 1 of the flipped instruction was designed based on the three-stage flipped classroom design framework (Estes et al., 2014). After implementation of version 1, the first post-experiment survey was conducted to identify problems in the first round. Based on the feedback from the first round of studies, version 2 of the flipped instruction was developed on the basis of version 1. In the second round, version 2 of the flipped instruction was implemented. The same survey was conducted after the second experiment to identify problems in the second experiment. Based on the feedback from the second round of research, version 3 of the flipped instruction was developed on the basis of version 2. In the last round, version 3 of the flipped instruction was implemented, and the post-experiment survey was also conducted. Version 3 is displayed in Figure 2.

### Procedure

The course was taught once a week with each class lasting 90 min. The learning workflow was divided into three stages: (1) Before class, the teacher designed the teaching schedule according to the teaching objectives, and made micro-videos using Camtasia Studio 9. The average duration was less than 10 min, which is in line with the optimal duration of recorded lectures (Hartley and Cameron, 1967; MacManaway, 1970). Then, the videos and learning materials together with some exercises were uploaded to the QQ learning platform for students’ autonomous learning. Students could put forward questions and ask for help on the QQ discussion platform when encountering difficulty if necessary. (2) In class, the teacher activated students’ prior knowledge by recalling relevant concepts or knowledge to create a clear contextual connection between the extracurricular and in-class learning. Students engaged in the in-class activities in groups. They then analyzed the problems encountered before class. Each group had a team leader to summarize and put forward questions they could not solve. The teacher explained the common difficulties in detail, and guided students to integrate the important knowledge of the lesson. Students then carried out individual exercises, and the teacher provided personalized instruction for students. (3) After class, students completed the exercises assigned by the teacher. When confronted with problems, students could repeatedly watch the micro video or communicate with the teacher and classmates on the learning platform. At the end of each class, students were required to submit a summary and reflection report on the QQ platform. The teaching plan was adjusted based on students’ reflection reports.

This course module lasted for 8 weeks. In the first and second weeks, classroom observation was conducted to understand the classroom status, and students finished the pre-questionnaire. Students completed pre-class, in-class, and after-class activities for the first topic lesson. Then, students experienced a three-round instructional process. In the third and fourth weeks, version 1 of the flipped instruction was implemented in the first round, and students completed pre-class, in-class, and after-class activities for the second topic lesson. The first post-experiment survey (classroom observation) was conducted to identify problems in the first round. In the fifth and sixth weeks, version 2 of the flipped instruction was implemented in the second round, and students completed pre-class, in-class, and after-class activities for the third topic lesson. The same survey was conducted after the second experiment. In the seventh and eighth weeks, version 3 of the flipped instruction was implemented in the third round, and students completed the activities for the fourth topic lesson. The post-experiment survey was also conducted. Thus, four classroom observations were implemented.

Table 2 summarizes each design element and justification for the flipped instruction design changes made after each iteration. As shown in Table 1, five major elements changed in the iteration: scaffolding, review pre-class learning, group collaboration, teacher’s feedback, and self-reflection.

**TABLE 2:** An overview of the three-round iteration of flipped instruction.

Iteration	Design element	Design description	Rationale	Feedback
First iteration	Scaffolding	Provided the key points and goals of video learning	Helped students to learn better autonomously	Video materials could not fully meet students’ learning needs
	Review pre-class learning	Reviewed video learning materials and solved problems	Encouraged students to prepare lessons actively	The class was passive
	Group collaboration	Communicated and solved problems in groups	Promoted students’ sense of belonging and cognitive engagement	Lack of interaction among group members
	Teacher’s feedback	Provided timely/constructive feedback	Supported students’ sense of competence and thus promoted their cognitive engagement	Some students did not get effective feedback from teachers
	Self-reflection	Uploaded the results of summary and reflection to the QQ platform	Improved students’ learning performance and motivation	The depth of students’ reflection was insufficient

Second iteration	Scaffolding	Provided supplementary learning materials	Met the needs of students’ autonomous learning	Learning materials did not meet individual needs
	Pre-class review	Used warm-up exercises before class	Promoted students’ cognitive engagement	Some students were still inattentive
	Group collaboration	Used inter-group competition activities	Improved student interaction	There were still marginal members who did not participate or contribute
	Teacher’s feedback	Patrolled between groups and provided individual feedback and guidance	Provided targeted feedback to students	Some students did not express their questions to the teacher
	Self-reflection	Uploaded the works to the QQ platform and reflected on their own performance by comparing with their peers’ work	Promoted students to perform better self-reflection	The summary and reflection uploaded by the students did not get the teacher’s response

Third iteration	Scaffolding	Provided a variety of learning materials	Met the different learning needs of students	Students’ autonomous learning needs were basically met
	Pre-class review	Added gamification to the warm-up exercises	Attracted the students’ attention	Most of the students responded positively
	Group collaboration	Students took turns as the group leader	Enhanced individuals’ sense of responsibility in group tasks	The balance of student participation improved significantly
	Teacher’s feedback	Reached out to as many students as possible and took the initiative to ask students about their learning	Provided timely teaching assistance to students	Most of the students received feedback from the teacher
	Self-reflection	The teacher shared and praised some of the students’ good summaries and reflections	Supported students’ cognitive and emotional engagement	Students were more positive in their self-reflections

### Data Analysis

SPSS 20.0 was adopted to analyze the experimental results in each design phase, including classroom observation and learning engagement questionnaire. Classroom observations were implemented in each round of the iterative experiment to analyze the effectiveness of flipped instruction for students’ learning engagement. Before and after the whole experiment, each student completed a pre- and post-intervention learning engagement questionnaire. Descriptive statistics were calculated. ANOVA and paired samples *t* tests were used to compare the pre- and post-intervention means.

## Results

### Analysis of Classroom Observation

Tables 3, 4 respectively show the ratings of students’ learning engagement in the four topic lessons based on classroom observations. Three rounds of iterative experiments were carried out. Before the first round of experiments, students’ learning engagement in the first topic lesson was rated through classroom observations. Therefore, the score of the first topic lesson was used as the starting point of the first round of experiments. A presentation order effect was found (see Table 4): behavioral engagement, *F*(3, 12) = 60.77, *p* = 0.000; emotional engagement, *F*(1.96, 3.93) = 262.99, *p* = 0.000; and cognitive engagement, *F*(1.04, 2.08) = 65.31, *p* = 0.013.

**TABLE 3:** Descriptive statistical results of classroom observation ratings of students’ learning engagement.

	Engagement	*N*	*Max*	*Mini*	*M*	*SD*
1st CO	Behavioral	5	3.47	3.27	3.37	0.09
	Emotional	3	3.47	3.27	3.36	0.10
	Cognitive	3	3.27	3.13	3.18	0.08
2nd CO	Behavioral	5	3.67	3.40	3.53	0.11
	Emotional	3	3.60	3.47	3.53	0.07
	Cognitive	3	3.40	3.27	3.31	0.08
3rd CO	Behavioral	5	3.80	3.53	3.70	0.10
	Emotional	3	3.80	3.60	3.69	0.10
	Cognitive	3	3.53	3.47	3.51	0.03
4th CO	Behavioral	5	4.00	3.86	3.94	0.06
	Emotional	3	4.00	3.80	3.91	0.10
	Cognitive	3	3.80	3.67	3.73	0.07

*CO, classroom observation.*

**TABLE 4:** Summary table for the one-way repeated measures ANOVA.

	Behavioral engagement	Emotional engagement	Cognitive engagement
F	*F*(3, 12) = 60.77, *p* = 0.000	*F*(1.96, 3.93) = 262.99, *p* = 0.000	*F*(1.04, 2.08) = 65.31, *p* = 0.013
1st – 2nd CO (F)	*F*(1, 4) = 56.61, *p* = 0.002	*F*(1, 2) = 602.14, *p* = 0.002	*F*(1, 2) = 97.50, *p* = 0.010
2nd – 3rd CO (F)	*F*(1, 4) = 80.87, *p* = 0.001	*F*(1, 2) = 172.97, *p* = 0.006	*F*(1, 2) = 41.59, *p* = 0.023
3rd – 4th CO (F)	*F*(1, 4) = 48.81, *p* = 0.002	*F*(1, 2) = 121.00, *p* = 0.008	*F*(1, 2) = 91.61, *p* = 0.011

The results showed that after the first round of instruction, the overall behavioral engagement (*M* = 3.53, *SD* = 0.11), emotional engagement (*M* = 3.53, *SD* = 0.07), and cognitive engagement (*M* = 3.31, *SD* = 0.08) in the second topic lesson were significantly better than the behavioral engagement (*M* = 3.37, *SD* = 0.09, *p* = 0.002 < 0.05), emotional engagement (*M* = 3.36, *SD* = 0.10, *p* = 0.002 < 0.05), and cognitive engagement (*M* = 3.25, *SD* = 0.71, *p* = 0.01 < 0.05) in the first topic lesson. Therefore, according to the classroom observation results of the round of experiments, the improved flipped instruction promoted students’ learning engagement. Then, after the second round of experiments, the results showed that the overall behavioral engagement (*M* = 3.70, *SD* = 0.10), emotional engagement (*M* = 3.69, *SD* = 0.10), and cognitive engagement (*M* = 3.51, *SD* = 0.03) in the third topic lesson were significantly better than the behavioral engagement (*M* = 3.53, *SD* = 0.11, *p* = 0.001 < 0.05), emotional engagement (*M* = 3.53, *SD* = 0.07, *p* = 0.006 < 0.05), and cognitive engagement (*M* = 3.31, *SD* = 0.08, *p* = 0.023 < 0.05) in the second topic lesson. Similarly, it showed that the improved flipped instruction was beneficial for students’ learning engagement. After the third round of experiments, the results of the classroom observation showed that behavioral engagement (*M* = 3.94, SD = 0.06, *p* = 0.002 < 0.05), emotional engagement (*M* = 3.91, *SD* = 0.10, *p* = 0.008 < 0.05), and cognitive engagement (*M* = 3.73, *SD* = 0.73, *p* = 0.011 < 0.05) in the fourth topic lesson were significantly better than those in the third topic lesson. Therefore, according to the classroom observation results of the third round of experiments, the continuously improved flipped instruction promoted students’ learning engagement.

### Analysis of the Learning Engagement Questionnaire

According to Tables 5, 6, the results of learning engagement reported by students showed that after three rounds of iterative experiments, students’ overall behavioral engagement (*M* = 4, *SD* = 0.25, *p* = 0.000 < 0.05), emotional engagement (*M* = 3.84, *SD* = 0.3, *p* = 0.00 < 0.05), and cognitive engagement (*M* = 3.82, *SD* = 0.19, *p* = 0.000 < 0.05) significantly improved. Importantly, the cognitive engagement of students was greatly promoted. The results show that the implementation of flipped instruction in this study was effective in terms of promoting students’ learning engagement.

**TABLE 5:** Descriptive statistical results of the learning engagement questionnaire.

	Engagement	*N*	*Max*	*Mini*	*M*	*SD*
Pre-intervention survey	Behavioral	10	3.86	3.25	3.52	0.21
	Emotional	8	3.53	2.78	3.13	0.24
	Cognitive	7	3.28	2.92	3.08	0.14
Post-intervention survey	Behavioral	10	4.31	3.47	4.00	0.25
	Emotional	8	4.11	3.17	3.84	0.30
	Cognitive	7	4.00	3.42	3.82	0.19

**TABLE 6:** Paired samples test of the learning engagement questionnaire.

Paired differences									

	Engagement	*M*	*SD*	Std. error mean	95% Confidence interval of the difference		*t*	df	Sig. (2 – tailed)
					
					Upper	Lower			
Pre- and post-intervention survey	Behavioral	−0.475	0.148	0.048	−0.581	−0.369	−10.172	9	0.000
	Emotional	−0.715	0.206	0.073	−0.887	−0.543	−9.811	7	0.000
	Cognitive	−0.743	0.152	0.057	−0.883	−0.603	−12.962	6	0.000

## Discussion

### The Influence of Three Rounds of Revision of Flipped Instruction on the Students’ Learning Engagement

Three versions of flipped instruction were applied in the same course in this study. The results showed that the revision of the flipped instruction promoted students’ learning engagement (behavioral, cognitive, and emotional) through the three rounds of iterative experiments. In terms of process assessment, classroom observation was used to score the learning engagement of the four topic lessons. Data analysis showed that under the guidance of each round of modified flipped instruction, students’ learning engagement in each topic lesson was significantly higher than in the previous lesson. In terms of summative assessment, pre-intervention and post-intervention questionnaire surveys were used to collect data. The results showed that students’ learning engagement was significantly improved after the experiment. Differing from many previous studies, this study discussed the three dimensions of students’ learning engagement. Bond (2020) pointed out that most studies tend to focus on learning engagement from one dimension, or have no clear definition of learning engagement. According to the results of this study, the revised flipped teaching significantly improved the three dimensions of students’ learning engagement, and especially their cognitive engagement.

### The Principles of Designing Effective Flipped Instruction for the Modern Educational Technology Course

From the three rounds of iterative experiments, according to the results, this study identified five design principles for effective flipped instruction in the MET course to promote students’ learning engagement, namely pre-class learning scaffolding (Principle 1), pre-class review (Principle 2), group collaboration (Principle 3), teacher’s feedback (Principle 4), and student reflection (Principle 5). The constructivism theory and individualized instruction theory are the backbone of the flipped instruction process.

For Principle 1, pre-class learning scaffolding should be provided. Pre-class learning activities are important for the success of flipped classrooms, as they facilitate students’ face-to-face learning engagement (Rahman et al., 2015; Yilmaz and Baydas, 2017). This study reveals that pre-class learning scaffolding is more conducive to improving students’ learning engagement in class. Two scaffolds were emphasized in this study to support students’ pre-class learning: provide key points and goals, and supplement various learning materials. According to the results of the classroom observation, students was able to respond quickly to the content of the pre-class learning materials when they mastered them with the support of the teacher. What’s more, the materials and discussion were related to clear learning objectives. The findings are coherent with those of Sergis et al. (2018), implying that the scaffolding provided by teachers helps students by making them more confident in engaging in the challenges of learning. Moreover, previous studies denoted that providing students with more autonomy to satisfy their personalized learning can increase students’ learning engagement (Rotgans and Schmidt, 2011; Lee et al., 2015). This perspective was also verified in the present study. From the perspective of individualized instruction theory, the types of learning materials are constantly diversified, providing students with more choices for personalized learning.

For Principle 2, pre-class review should be conducted. In terms of pre-class review, it was found that the game elements designed in the exercise can promote students’ learning engagement. When the points system and leaderboards were added in the pre-class review activities, classroom observation showed that the class as a whole changed from passive to active, and most of the students exhibited attentive body language. Moreover, many students performed enthusiasm and interest. These findings have been reported in previous studies (Hew et al., 2016; Jo et al., 2018; Lo et al., 2018; Huang et al., 2019; Ninaus et al., 2019; Zainuddin et al., 2020). Zamzami (2018) explained that students feel more competent in gamified flipped classrooms because pre-class learning gives them more opportunities to master their learning and to earn points through gamified competitive activities. From the perspective of the constructivism theory, the gamified exercises embody the idea of student-centeredness and make students more willing to engage in the class.

For Principle 3, group collaboration should be effectively organized. The current study adopted competitive activities and rotation of group leaders to promote group collaboration. It successfully supported students to benefit from it and to improve their learning engagement. The classroom observations showed that the interaction between group members increased significantly when the competition between groups and the rotation of group leaders were involved. From the perspective of the constructivist learning theory, learning is constructed by groups, not just individuals (Alonso et al., 2005). Group collaborative learning can create an environment in which students interact with peers and experience social relatedness (Abeysekera and Dawson, 2015). However, this study found that some students in group cooperation still had a low degree of learning engagement. Classroom observation showed that there were still some marginal members who did not participate or contribute. This may have something to do with grouping (Zheng et al., 2019). Dividing into groups heterogeneously is very important for group cooperation, in accordance with the Zone of Proximal Development theory proposed by Vygotsky (1978). Students should be divided into groups according to their subject matter knowledge and sociocultural background.

For Principle 4, teacher’s feedback should be given in time. It was found in this study that timely and effective teacher feedback can promote students’ learning engagement. From the classroom observation, it could be seen that students showed enthusiasm and interest when teachers provided individualized feedback through inter-group patrols and active attention. This also echoed previous studies (Niemiec and Ryan, 2009; Abeysekera and Dawson, 2015; Lucena et al., 2020). From the perspective of constructivism and individualized instruction theory, teachers should not only assist students to construct knowledge, but also provide personalized teacher support tailored to students’ different demands. Teacher feedback creates conditions for teacher-student interaction and encourages students to actively engage in learning. Teachers’ active attention can also make students feel emotional support from teachers, which is an important way to promote students’ emotional engagement (Rimm-Kaufman et al., 2014).

For Principle 5, student reflection should be valued. This study revealed that self-reflection plays an important role in promoting students’ learning engagement. The classroom observation showed that students performed better learning engagement when they were engaged in self-reflection. Self-reflection provides students with an opportunity to examine their own learning, which is conducive to improving the learning effect and facilitating students’ learning engagement in flipped classrooms (Stefano et al., 2016; Chen et al., 2019). Peer comparison and teacher feedback were used in this study to support students’ self-reflection. Students’ feedback indicated that they exhibited better self-reflection when comparing their peers’ works. Especially for skills training courses such as MET, students can find their own problems and improve their operational skills by comparing them with their peers (Lin et al., 2021). From the results of the observations, students were more positive in their self-reflections when the teacher shared and praised some of the students’ good summaries and reflections. Teachers’ encouragement and praise provide students with emotional teaching support and can stimulate students’ learning engagement (Ruzek et al., 2016). However, most of the previous studies focused on students’ behavioral engagement (Wang, 2017, 2019). This study further explored how to support students’ emotional and cognitive engagement through self-reflection.

## Conclusion

Based on the theoretical framework of learning engagement proposed by Fredricks et al. (2004), this study explored how to promote pre-service students’ learning engagement in the flipped classroom. Combined with process assessment and summative assessment, classroom observation and questionnaires were used to evaluate students’ learning engagement. This study found the effectiveness of flipped instruction in terms of students’ learning engagement in the MET course with five principles, and the active influence of three rounds of revision of flipped instruction on the students’ learning engagement.

### Implications

This study used the DBR approach to explore how to promote pre-service teacher students’ learning engagement in the flipped MET course. Several principles were developed through three rounds of iterative experiments. These principles are as follows: (1) provide key points and supplement various learning materials for pre-class learning, (2) review students’ pre-class learning in gamified warm-up exercises, (3) promote group cooperation using competitive activities and rotation of group leaders, (4) provide timely and effective teacher feedback through inter-group patrols and active attention, and (5) promote students’ self-reflection through peer comparison and teacher feedback. Therefore, this study not only improves the effectiveness of flipped classrooms for students’ learning engagement, but also provides an experience reference for teachers to design and conduct flipped instruction. In terms of theory, although there have been many studies on the relevance of flipped classrooms and learning engagement, there are few empirical studies which have discussed the promotion of students’ learning engagement based on this theory. This study advances the understanding of the theories (students’ learning engagement and flipped classroom) while applying them. Therefore, this study extends existing research on the role of flipped classrooms in learning engagement, including behavioral, cognitive, and emotional engagement.

### Limitations and Future Work

However, this study still has some limitations based on which some suggestions for future research are suggested. Firstly, the time between iterations was short, leaving little time for in-depth analysis between iterations, which could have led to ill-considered instant decisions. Secondly, this study was carried out in only one university, and the sample was limited to pre-service teacher students. Any particular cycle of DBR studies is context-specific. Therefore, the results of the research should be considered with caution. These limitations require that future work extend the iteration cycle and the sample for more comprehensive findings. In addition, interviews along with other instruments should be added to future studies to further support the results. Finally, the proposed design principles just apply to MET courses. Therefore, further research is needed to test the design principles in other courses related to operational skills training, such as medical education and physical education.

## Data Availability Statement

The original contributions presented in the study are included in the article/supplementary material, further inquiries can be directed to the corresponding author.

## Ethics Statement

Ethical review and approval were not required for the study on human participants in accordance with the local legislation and institutional requirements. Written informed consent for participation was not required for this study in accordance with the national legislation and the institutional requirements.

## Author Contributions

All authors contributed equally to the conception of the idea, implementing and analyzing the experimental results, writing the manuscript, and reading and approving the final manuscript.

## Conflict of Interest

The authors declare that the research was conducted in the absence of any commercial or financial relationships that could be construed as a potential conflict of interest.

## Publisher’s Note

All claims expressed in this article are solely those of the authors and do not necessarily represent those of their affiliated organizations, or those of the publisher, the editors and the reviewers. Any product that may be evaluated in this article, or claim that may be made by its manufacturer, is not guaranteed or endorsed by the publisher.
